# The relationship between *NQO1 *C609T and *CAT *C-262Tgenetic polymorphisms and the risk of age-related cataracts

**Published:** 2015-09

**Authors:** Narjes Zarei, Iraj Saadat, Majid Farvardin-Jahromi

**Affiliations:** 1 Department of Biology, College of Sciences, Shiraz University, Shiraz, Iran; 2 Department of Ophthalmology, Shiraz University of Medical Sciences, Shiraz, Iran

**Keywords:** Cataract, *CAT*, Genetic polymorphism, *NQO1*

## Abstract

Cataract is multi-factorial eye disease identified by the disturbance of the transparent ocular lens. There is significant evidence suggesting oxidative damage as a major cause of initiation and progression of numerous diseases including cataracts. NAD(P)H:quinone oxidoreductase 1 (NQO1; OMIM: 125860) and catalase (CAT, OMIM: 115500) are antioxidant enzymes that prevent cells from oxidative stress. The aim of the present study was to investigate the association between *NQO1 C609T *(Pro189Ser, rs1800566) and *CAT *promoter *C-262T *(rs1001179) genetic polymorphisms and the susceptibility to cataracts. A case-control study including 190 cataracts cases and 190 healthy subjects was carried out. Genotype distributions of *NQO1 *and *CAT *polymorphisms were examined using polymerase chain reactions and a restriction fragment length polymorphism (PCR-RFLP) approach to investigate the possible role of these polymorphisms as risk factors in the development of cataracts. Variant CT heterozygous and TT genotypes of the *NQO1 C609T *polymorphism were found to be associated with an increased risk of cataracts (CT *vs *CC, OR=1.61, 95%CI: 1.02-2.52, P=0.038), (CT/TT *vs *CC, OR=1.56, 95%CI: 1.02-2.4, P=0.040). In addition, compared to indoor work places and the CC genotype of *NQO1, *outdoor work places and CT/TT genotypes of *NQO1 *were found to increase the risk of age-related cataracts (OR=2.75, 95%CI: 1.20-6.33, P=0.017). The analysis did not reveal, however, any statistically significant (P>0.05) difference between *CAT C-262T *polymorphism and the risk of cataracts.

## INTRODUCTION

Lens is one of the most essential parts of the eye which is susceptible to oxidative damage [1]. Since lens’ fiber cells are not renewed and have to last for a lifetime, damage to these cells can result in protein degradation, ultimately causing age-related cataracts [1, 2]. Cataract is ranked as the leading cause of blindness (51%) worldwide.

Exposure to sunlight and ultraviolet B light trigger the generation of reactive oxygen species and free radicals, causing age-related cataracts [3].

Hence, the eye lens has developed a wide variety of protective and repair systems to defend itself against oxidative stress. NAD(P)H:quinone oxidoreductase 1 (NQO1; OMIM: 125860) is a cytosolic enzyme (a phase II enzyme) which catalyzes two- electron reduction quinones, quinonimins, nitroaromatic and azo dyes and prevents the generation of semiquinone free radicals and reactive oxygen species, thus protecting cells from oxidative damage [4-8]. In addition to its catalytic role in quinones, NQO1 has been reported to show superoxide scavenging activity [9]. Studies have confirmed that the single C to T substitution at nucleotide 609 (rs1800566) decreased the enzyme activity of NQO1 [10]. Catalase (CAT, OMIM: 115500), which is in the first line of cellular defense against reactive oxygen species, is another antioxidant enzyme that metabolizes H2O2. C-262T genetic polymorphism (rs1001179) exists in the promoter region of the human *CAT *gene [11].

As previously reported, genetic polymorphisms in the *GSTM1 *and *GSTO2 *detoxification enzymes increased the risk of developing cataract in outdoor work places [12]. Genetic variations in the antioxidant genes coding for the CAT and NQO1 enzymes may lead to decreased or impaired enzymatic activity and alter ROS detoxification. Therefore, genetic variations of enzymes that protect cells against ROS may modulate cataract risk. To the authors’ knowledge, no study has yet investigated the additive effect of these polymorphisms (*NQO1 *C609T and *CAT *C-262T) as related to outdoor work places (as an indicator of exposure to sunlight) on the risk of cataracts. The present case-control study was performed, therefore, to achieve this goal**.**

## MATERIALS AND METHODS

**Subjects: **This study included 190 patients with senile cataracts, recruited from Khalili and Pars Hospitals, Shiraz, Iran. We excluded patients with secondary cataracts caused by diabetes, trauma, steroid administration, and other origins. The Iranian population is reported to be one of the most heterogeneous [13, 14]; therefore, patients and controls were selected from the same ethnic, religious group (Persian Muslims living in Fars province, southern Iran). The mean age of the cataract patients and the controls was 66.59±11.6 years and 58.41±8.9 years, respectively. Age distribution was significantly different between the cases and the controls (t=7.62, df=373, P=0.015). The participants were divided into two groups according to their occupation: outdoor workers who were exposed to sunlight (farmers, drivers, etc) and indoor workers, who were not exposed to direct sunlight (housewives, teachers, etc.). The study was approved by the local Bioethics Committee of Shiraz University, and each patient gave written informed consent before being enrolled in the study.

**Figure 1:**
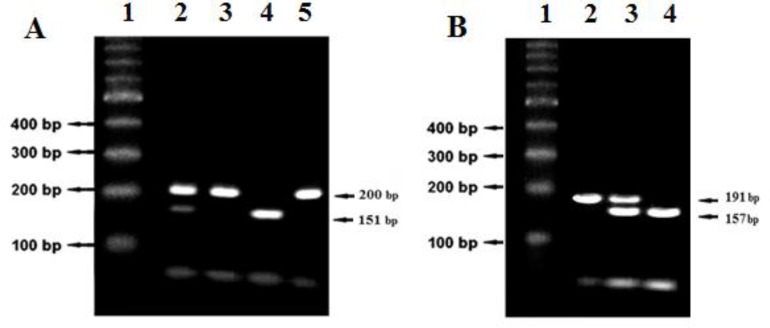
Genotyping of the *NQO1* C609T (A) and *CAT* C-262T (B) polymorphisms by *HinfI* and *EcoRV* RFLP. **A)** From left to right the lanes are 1) DNA size marker (100 bp ladder), 2) CT genotype (200, 151 and 49 bp), 3) CC genotype (200 bp), 4) TT genotype (151 and 49 bp) and 5. intact (200 bp) **B)** From left to right the lanes are 1) DNA size marker (100 bp ladder), 2) TT genotype (191 bp), 3) TC genotype (191, 157 and 34 bp) and 4) CC genotype (157 and 34 bp

**DNA extraction and genotyping: **Five ml venous blood was collected from all of patients and controls in EDTA tubes. DNA extraction was performed using standard methods [15]. The primers used to determine *NQO1 *and *CAT *genotypes are shown in Table 1 [4, 16]. For RFLP, PCR products of *NQO1 *C609T and *CAT *C–262Tpolymorphisms were digested with *HinfI *(5 U at 37°C for 16 h) and *EcoRV *(5 U at 37°C for 16 h) (Fermentas), respectively. For the *NQO1 *C609T polymorphism, CC was identified by a single band (200 bp), CT was identified by three bands (200, 151 and 49 bp) and the hemozygous TT variant displayed two bands (151 and 49 bp). As per the *CAT*–262 C/T polymorphism, TT was identified by a single band (191 bp), TC was identified by three bands (191, 157 and 34 bp) and the hemozygous CC variant displayed two bands (157 and 34 bp) (Fig. 1).

**Table 1:** Primers used for genotyping of *NQO1* and *CAT* genes

**Primers**	**Primer sequence (5’-3’)**	**Product size ** **(bp)**	**Melting temperature ** **(°C)**
***NQO1 *** **(F)**	TCCTCAGAGTGGCATTCTGC	230	58
***NQO1 *** **(R)**	TCTCCTCATCCTGTACCTCT		
***CAT *** **(F)**	CTGATAACCGGGAGCCCCGCCCTGGGTTCGGATA	191	68
***CAT *** **(R)**	CTAGGCAGGCCAAGATTGGAAGCCCAATGG		

**Statistical analysis: **An *x*2 test was used for *NQO1 *and *CAT *polymorphisms to determine whether control samples demonstrated the Hardy–Weinberg equilibrium. Unconditional logistic regression was used to calculate ORs and 95% CI for cataracts’ risk associated with the genetic polymorphisms of *NQO1 *and *CAT*. Considering the significant age difference between the patients and the controls, logistic regression was used in further analyses to calculate ORs and 95%CI for various genotypes after adjusting for age. Statistical analysis was performed using the Statistical Package for Social Sciences (SPSS Inc. version11.5; Chicago, IL.). A probability of P<0.05 was considered as statistically significant.

## RESULTS AND DISCUSSION

The genotypic distributions of *NQO1 *C609T and *CAT *C-262T polymorphisms in the cataracts patients and the controls are shown in Table 2. The *NQO1 *and *CAT *genotypes in the control subjects followed the Hardy-Weinberg equilibrium (For *NQO1 *C609T polymorphism: *x*2=2.12, df=1, P=0.145; For *CAT *C-262T polymorphism: *x*2=0.02, df=1, P=0.897).

No significant relationships were observed between genetic polymorphism of *CAT *and risk of cataracts, even after age adjustment (Table 2). By comparing the frequency of the *NQO1 *genotypes among cases and controls it was revealed that the CT heterozygous and the combination of the CT (OR=1.61, 95%CI: 1.02-2.52, P=0.038) and CT+TT (OR=1.56, 95%CI: 1.02-2.4, P=0.040) genotypes compared with the CC increased susceptibility to cataracts.

**Table 2:** Associations between genetic polymorphisms of *NQO1* C609T and *CAT* C-262T and risk of cataract

**Polymorphisms**	**Controls**	**Cases**	**OR**	**95%CI**	**P**	**OR** *	**95%CI**	**P**
***CAT *** **C-262T**								
**CC**	127	127	1.0	-	-	1.0	-	-
**CT**	58	57	1.02	0.66-1.58	0.938	1.13	0.70-1.82	0.620
**TT**	5	6	0.83	0.25-2.80	0.768	0.93	0.26-3.37	0.913
								
**NQ01 C609T**								
**CC**	116	135	1.0	-	-	1.0	-	-
**CT**	65	47	1.61	1.02-2.52	0.038	1.86	1013-3.04	0.014
**TT**	9	8	1.30	0.48-3.50	0.590	1.35	0.46-4.02	0.850
**CT+TT vs CC **	74	55	1.56	1.02-2.40	0.040	1.78	1.11-2.85	0.016

* **Note: **Adjusted OR for age of participants.

Among our participants 58 and 31 of the cataract patients and control subjects worked outdoor, respectively. The compression of work place between cataracts patients and the control individuals was statistically significant, revealing an increasing susceptibility to cataracts for those working outdoors (OR=1.67, 95%CI: 1.01-2.77, P=0.044). It should be mentioned that information on work place was missing for 6 patients and 46 controls.

Given that cataracts associated with the work place in the present study, the analysis was performed to investigate the additive effects of genotype and work place. Table 3 shows the *NQO1 *polymorphism of cases and controls as related to work place. The reference group consisted of individuals who worked indoors and had the CC genotype (putative as low risk condition). The analysis shows that the risk of cataracts increased when the two risk factors (outdoor work place and the CT/TT genotypes) were considered together (OR=2.75, 95%CI: 1.20-6.33, P=0.017). The results of these analyses can be challenged, however, because data on the work place, which is known to be associated with the risk of cataracts, was missing for some patients and control subjects.

**Table 3:** The association between *NQO1* C609T polymorphism, work place and cataract risk

Work place	*NQO1* genotypes	Patients	Controls	Number of risk factors	OR	95%CI	P
Indoor	CC	76	82	0	1.0	-	-
Indoor	CT+TT	50	31	1	1.74	1.01-3.00	0.047
Outdoor	CC	35	22	1	1.72	0.925-3.18	0.087
Outdoor	CT+TT	23	9	2	2.75	1.20-6.33	0.017

To summarize, no association was found between the genotypes of *CAT *C-262T polymorphism and risk of cataracts. The results of this study are consistent with previous investigations which showed that the *NQO1 *T allele increased susceptibility to various cancers such as lung cancer [17], leukemia [18], cutaneous basal cell cancer [19], colorectal cancer [4, 20], bladder cancer [21] and ovary cancer [22].

Since the frequency of the TT genotype of the *NQO1 *C609T polymorphism was low in the current study (4.7 and 4.2% in the patients and healthy controls, respectively) the statistics might not be sufficient to detect the weak effect of this variant genotype on the risk of cataracts. Further studies with larger sample sizes are thus warranted. The underlying mechanism of the correlation between *NQO1 *C609T polymorphism and increased risk for developing cataracts may be related to different enzyme activities encoded by different *NQO1 *genotypes. Thus, reduced *NQO1 *activity caused by C to T substitutions can result in reduced detoxification, leading cells to be easily damaged by ROS, thereby increasing the susceptibility to senile age-related cataracts.

## Notes

**Acknowledgements: **The authors are indebted to the participants for their close cooperation. This study was supported by Shiraz University.
